# Remote consultations in prison mental healthcare in England: impacts of COVID-19

**DOI:** 10.1192/bjo.2021.13

**Published:** 2021-02-08

**Authors:** Thomas Hewson, Louise Robinson, Najat Khalifa, Jake Hard, Jennifer Shaw

**Affiliations:** Academic Clinical Fellow in Psychiatry at the Health Education North West School of Psychiatry and the University of Manchester, UK; Consultant Forensic Psychiatrist Lancashire and South Cumbria NHS Foundation Trust and an Honorary Senior Lecturer in forensic psychiatry in the Division of Psychology and Mental Health at the University of Manchester, UK; Associate Professor in Forensic Psychiatry in the Department of Psychiatry and Centre for Neuroscience Studies at Queen's University, Kingston, Ontario, and regional psychiatry lead (Ontario) in Correctional Service Canada; General Practitioner with over 13 years of experience working in prisons in the UK and is the Chair of the Royal College of General Practitioners Secure Environments Group, London, UK; Professor of Forensic Psychiatry in the Division of Psychology & Mental Health at the University of Manchester, UK, Consultant Forensic Psychiatrist and academic lead for the Offender Health Research Network. She is also a member of the Independent Advisory Panel on Deaths in Custody, UK.

**Keywords:** Forensic mental health services, telepsychiatry, prison, COVID-19, coronavirus

## Abstract

Telemedicine has become increasingly used by prison mental health services throughout the COVID-19 pandemic. In this editorial, we explore the benefits and risks of the remote provision of forensic mental healthcare, with consideration of the clinical, financial, ethical and legal consequences.

Telemedicine is the delivery of healthcare services using information and communication technologies for the exchange of information to diagnose, treat and prevent illness and injuries, and for the purposes of health research and health education.^1^

Prior to the COVID-19 pandemic, 50 of the 117 prisons in England and Wales lacked sufficient internet connectivity for videoconferencing, and only two telemedicine solutions were approved for use in Her Majesty's Prison and Probation Service (HMPPS).^2^ Following COVID-19, new legislation permitted the use of 4G-enabled tablets for telemedicine within secure environments.^2^ Despite initial delays in implementing this technology, all prisons in England now have telemedicine capability.^2^

## Infection control

Conducting healthcare consultations remotely aids infection control by allowing compliance with social distancing, a key element of the strategy to protect prisoners from coronavirus infection. The flow of persons into and out of prisons and face-to-face contact between clinicians, prison staff and patients are reduced. This is important given the high population density of prisons, which increases risk of explosive outbreaks of infectious diseases. Furthermore, compared with the general population, prisoners experience higher rates of multi-morbidity,^3^ predisposing them to greater risk of mortality from infection. For these reasons, measures to minimise disease transmission may persist longer in prisons than in wider society. The use of technology to facilitate the mental health assessment and treatment of prisoners remotely is in keeping with these considerations. The prospect of future infectious disease outbreaks suggests a potential for telemedicine to safeguard the delivery of future healthcare services.

## Accessibility of healthcare services

Implementation of telemedicine services in prison settings should take into consideration barriers to access relating to security measures, which are more stringent in higher than lower levels of security, and the unpredictable nature of the prison environment, where security often takes priority over the provision of healthcare. Individual patients may be unable to access telemedicine services owing to person-specific security considerations, requiring alternative options for healthcare engagement.^4^

Telemedicine could increase the accessibility of prison healthcare. Owing to security procedures, escorting prisoners to appointments with mental health services can present challenges that could be addressed by remote healthcare. Reduced visibility of appointments could also reduce stigma that prisoners may experience after seeing a mental health clinician. Access to healthcare services may additionally be improved through prison-to-prison uses of telemedicine, whereby healthcare professionals practising in one prison could offer support to colleagues in others. This could immensely benefit smaller establishments where general practitioners and psychiatrists are not present throughout the entire working week.

## Diagnostic accuracy and reliability

Overall, the research literature supports the diagnostic accuracy and reliability of remote psychiatric assessments, with many studies demonstrating equivalence with face-to-face encounters.^5^ However, there is a significant lack of robust evidence especially in forensic psychiatry, with most data emerging from case reports, descriptive studies and uncontrolled trials.^5^ The literature also varies widely in its use of different telehealth technologies and in the types of psychiatric assessment conducted. Furthermore, most studies are international and utilise small samples. Consequently, the generalisability of their findings to English offender populations is uncertain. The risks of a weak evidence base for telepsychiatry in forensic services include the potential for poorer clinical care, missed or delayed diagnoses, inappropriate or ineffective treatment and safety risks for the public when considering the consequences of inaccurate risk assessments in criminal cases.

Specific concerns have been voiced regarding potential limitations of telemedicine in conducting psychiatric assessments. It has been suggested that clinicians may miss subtle but significant cues in their mental state examinations, such as body odour and signs of psychomotor agitation.^6^ Demonstrating empathy and establishing a strong rapport may be more difficult via video technology than in person.^6^ This is important given the frequent need to discuss sensitive topics such as suicidal and violent thoughts, self-harm, offending behaviour and previous trauma.

## Acceptability to patients and clinicians

Both patients and services are increasingly accepting of the use of video links in forensic mental healthcare.^7^ However, the acceptability of telepsychiatry may vary according to individual patients’ preferences, needs and underlying mental disorder(s). Tucker et al found that, although prisoners preferred face-to-face assessments, remote consultations were preferred for discussing difficult topics such as sexual abuse.^8^ Some patients mistrust technology in healthcare, particularly those with persecutory delusions. However, one review found little evidence of difficulties in conducting telehealth consultations with patients with psychosis, citing two clinical trials supporting the effectiveness and acceptability of such assessments in this patient population.^9^ Other groups that may require special considerations are those with intellectual disability and educational deficits, both of which are overrepresented in the criminal justice system;^10^ impaired concentration, attention and language abilities could hinder a person's ability to focus on a computer screen and to engage with remote assessments. Patients with sensory deficits may also face challenges, and clinicians need to ensure that telemedicine services are accessible for these individuals. The use of a healthcare chaperone in this context could prove very beneficial. More widely, the use of healthcare chaperones during virtual healthcare assessments could help patients in their operation of virtual technology and engagement with the care provider and could provide improved continuity of care; however, this represents an additional resource and cost. Education and training for staff and patients is also an essential component of implementing accessible and acceptable telehealth services and ensuring that both groups feel comfortable in utilising telehealth technology.^4^

The impacts of telemedicine on the feelings of patients should be considered. Some may feel empowered by the use of this technology and feel safe and secure knowing that they are able to access healthcare remotely. However, for others, the lack of face-to-face contact could make them feel further isolated, stigmatised and confined. To overcome such feelings of isolation, some prisons have been utilising secure video calling with family members throughout the pandemic, and temporary secure phone handsets have been issued to all prisons that previously did not have in-cell telephony installed.^11^

Reluctance of clinicians to engage with this technology, given some of the concerns raised above, remains a potential barrier to use of videoconferencing in forensic psychiatry. However, the coronavirus pandemic may have prompted an attitudinal shift for professionals and the public, as remote consultations have become more common throughout healthcare.^12^ Some clinicians may welcome the introduction of telepsychiatry as an opportunity to increase their private practice by conducting remote assessments for courts away from their local area. This may, however, lead to loss of the contribution of understanding of local services and pathways to such assessments.

## Patient safety

Management of patient safety during the consultation is another important consideration. Patients may, for example, engage in dangerous behaviours such as self-harm, and the clinician's ability to intervene quickly is more limited when consulting remotely. Carefully designed procedures to manage such situations and mitigate the risks are required. The physical presence of healthcare chaperones during remote consultations could be one method of lessening these risks, but individuals in these roles would require the appropriate training. The American Telemedicine Association has developed guidelines for clinicians dealing with emergencies that may arise when using videoconferencing,^13^ but such guidelines are currently lacking in England.

## Ethical and legal considerations

The principle of equivalence dictates that prisoners should have access to equitable healthcare and should not be disadvantaged compared with members of the general public. Edge et al recently coined the term ‘digital inequivalence’ to describe the lesser access to healthcare technology in prisons, highlighting that prisons were slower to adopt telehealth services at the start of the pandemic.^2^ Prisons face the complex task of balancing security and care. If society returns to face-to-face secondary care appointments, but prisons accelerate their use of telehealth and video consulting, this will raise important ethical questions regarding the above principle. The lack of a robust evidence base makes calculation of clinical equivalence challenging.

Issues regarding privacy, confidentiality, security and safety have been outlined in relation to the use of videoconferencing for forensic healthcare consultations.^6,7^ Clinicians may have concerns about safely using technology and managing data, demonstrating the need for appropriate information technology resources (secure networks and encryption programmes) and training. Although the Ministry of Justice and the National Health Service have specific criteria governing data protection and the use of information technology, there are no uniform regulations in England specifically governing the use of telepsychiatry, perhaps contributing to lack of professional confidence. However, aspects of confidentiality may be improved through telemedicine approaches, by reducing the need for prison officers to escort prisoners to healthcare appointments and thereby reducing patients' fears of other prison staff and prisoners being aware of their illness. It seems appropriate that the patient should be made aware of the risks and benefits of videoconferencing and give informed consent for its use.^7^ However, in the context of prison assessments, where there may be no alternatives and the patient may have little choice if they are to engage in the criminal justice process or receive healthcare, such consent may lack validity.

## Considerations of finance and efficiency

Remote assessments remove the requirement for travel to and from prisons. The costs of travel, including travel time, can be significant owing to the wide geographical spread of prisons throughout England, thus offering the potential for significant economic savings and improved efficiency.^7^ Given these time and direct cost savings, clinicians should be able to complete assessments in a timelier manner and, potentially, more frequently. This may be particularly useful in the current climate of delayed court proceedings due to the effects of the COVID-19 pandemic,^14^ potentially forming part of the wider strategic response to ensuring a rapid recovery of the judicial system. Furthermore, reducing the time spent waiting for assessments and clinical care would have a positive clinical impact, particularly if this reduced the time taken for emergency transfers to hospital under conditions of the Mental Health Act.

## Key patient-focused points

(a) Clinicians should assess the feasibility, safety and appropriateness of conducting forensic mental health consultations virtually when scheduling health appointments with their patients.
(b) A tailored approach should be considered, taking account of each patient's individual circumstances and the likely impact that such circumstances could have on effective remote healthcare engagement and delivery.
(c) Clinicians should pay particular attention to patient groups that may experience greater difficulties in engaging with remote assessments, and organisations must consider reasonable adjustments and alternative routes to care for these individuals.

## Conclusions

Remote consultations offer useful solutions for forensic psychiatric assessment and treatment during restricted prison regimes in which face-to-face assessments are not feasible. They may also offer benefits such as reduced costs and improved efficiency for prisons and mental health services. However, clinical equivalence has yet to be robustly demonstrated and risks of poorer health outcomes for prisoners remain. We recommend that research on the impacts, particularly on clinical outcomes, of remote psychiatric consultations in prisons is urgently conducted as well as further exploration of acceptability to prisoners. Clear clinical guidelines to address the unique risks for patient and professional are required; learning from countries more familiar with this practice, such as the USA, Canada and Australia, would support this work. These actions are required before remote consultations become accepted as routine in forensic psychiatry.
